# Genome-wide transcriptome analysis using RNA-Seq reveals a large number of differentially expressed genes in a transient MCAO rat model

**DOI:** 10.1186/s12864-018-5039-5

**Published:** 2018-09-05

**Authors:** Lyudmila V. Dergunova, Ivan B. Filippenkov, Vasily V. Stavchansky, Alina E. Denisova, Vadim V. Yuzhakov, Sergey A. Mozerov, Leonid V. Gubsky, Svetlana A. Limborska

**Affiliations:** 1 Human Molecular Genetics Department, Institute of Molecular Genetics, Russian Academy of Sciences, Moscow, Russian Federation; 2 Research Institute of Cerebrovascular Pathology and Stroke, Pirogov Russian National Research Medical University, Moscow, Russian Federation; 3 A. Tsyb Medical Radiological Research Center – branch of the National Medical Research Radiological Center of the Ministry of Health of the Russian Federation, Obninsk, Russian Federation

**Keywords:** tMCAO, RNA-Seq, Gene expression, mRNA, Neurotransmission, Inflammation

## Abstract

**Background:**

The transient middle cerebral artery occlusion (tMCAO) model is used for studying the molecular mechanisms of ischemic damage and neuroprotection. Numerous studies have demonstrated the role of individual genes and associated signaling pathways in the pathogenesis of ischemic stroke. Here, the tMCAO model was used to investigate the genome-wide response of the transcriptome of rat brain tissues to the damaging effect of ischemia and subsequent reperfusion.

**Results:**

Magnetic resonance imaging and histological examination showed that the model of focal ischemia based on endovascular occlusion of the right middle cerebral artery for 90 min using a monofilament, followed by restoration of the blood flow, led to reproducible localization of ischemic damage in the subcortical structures of the brain. High-throughput RNA sequencing (RNA-Seq) revealed the presence of differentially expressed genes (DEGs) in subcortical structures of rat brains resulting from hemisphere damage by ischemia after tMCAO, as well as in the corresponding parts of the brains of sham-operated animals. Real-time reverse transcription polymerase chain reaction expression analysis of 20 genes confirmed the RNA-Seq results. We identified 469 and 1939 genes that exhibited changes in expression of > 1.5-fold at 4.5 and 24 h after tMCAO, respectively. Interestingly, we found 2741 and 752 DEGs under ischemia–reperfusion and sham-operation conditions at 24 h vs. 4.5 h after tMCAO, respectively. The activation of a large number of genes involved in inflammatory, immune and stress responses, apoptosis, ribosome function, DNA replication and other processes was observed in ischemia–reperfusion conditions. Simultaneously, massive down-regulation of the mRNA levels of genes involved in the functioning of neurotransmitter systems was recorded. A Kyoto Encyclopedia of Genes and Genomes pathway enrichment analysis showed that dozens of signaling pathways were associated with DEGs in ischemia–reperfusion conditions.

**Conclusions:**

The data obtained revealed a global profile of gene expression in the rat brain sub-cortex under tMCAO conditions that can be used to identify potential therapeutic targets in the development of new strategies for the prevention and treatment of ischemic stroke.

**Electronic supplementary material:**

The online version of this article (10.1186/s12864-018-5039-5) contains supplementary material, which is available to authorized users.

## Background

Among the cerebrovascular diseases of the brain, ischemic stroke remains one of the leading causes of mortality and disability [1–3]. This disease occurs as a result of a critical reduction in blood flow in the brain tissue, which leads to the death of neurons and glial cells, accompanied by massive inflammation. The models of cerebral ischemia in laboratory animals are used to understand the processes underlying the damage and recovery of neurological functions after cerebral ischemic injury, as well as to study the protective properties of drugs [4–10]. The main strengths and weaknesses of the most commonly used animal models of acute ischemic stroke have been described in a number of review articles [11–13].

The model of transient middle cerebral artery occlusion (tMCAO) is widely used for the development of neuroprotective therapeutic approaches. tMCAO is based on temporal artery occlusion and subsequent restoration of blood flow. According to Howells et al., the model that was created using an intravascular filament was used in 42.2% of 2582 neuroprotection experiments [13]. The tMCAO model reflects events that occur in ischemic stroke in humans in the treatment of thrombolytic agents [14, 15]. The results of clinical studies indicate that, currently, thrombolysis is one of the most effective and affordable methods of treatment of ischemic stroke [16, 17]. Concomitantly, it is known that reperfusion after thrombolysis can enhance the damage caused by ischemia [18]. The brain damage caused by ischemia–reperfusion (IR) leads to the disruption of the functioning of multiple genes [19–21]. However, at present, most studies have analysed the features of the expression of a small number of genes associated only with one or a few signaling pathways [5, 22–25].

In the present study, we used high-throughput mRNA sequencing (RNA-Seq) to study the genome-wide response of the transcriptome to the damaging effect of IR in a tMCAO model based on endovascular artery occlusion (90 min) and subsequent reperfusion. The tMCAO model created using this duration and method of occlusion is one of the most commonly used models of rat cerebral ischemia [26–28]. We identified hundreds of differentially expressed genes (DEGs) and their functional associations in the rat brain sub-cortex, which included necrotic and penumbra areas under the tMCAO model conditions. These new data indicate a complex spatial-temporal genome regulation of brain cells under IR that can be used to identify potential therapeutic targets in the development of new strategies for the prevention and treatment of ischemic stroke.

## Results

### Characterization of ischemia using magnetic resonance imaging (MRI)

**Fig. 1:**
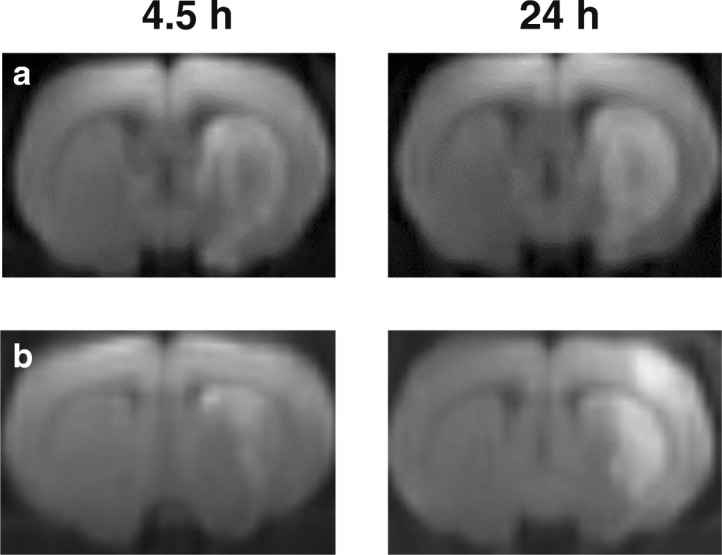
Characterization of ischemia using magnetic resonance imaging. A typical diffusion-weighted imaging (DWI) of the formation of ischemic injury areas with a subcortical localization both 4.5 and 24 h after tMCAO (**a**), as well as subcortical and hemispheric localization at 4.5 and 24 h after tMCAO, respectively (**b**) in the rat brain

Using the diffusion-weighted imaging (DWI) and T2-weighted imaging (T2 WI) modes of magnetic resonance imaging (MRI), we detected the location and volume of ischemic foci in animals after tMCAO. A typical the diffusion-weighted imaging (DWI) of the formation of ischemic injury areas with a subcortical (cerebral subcortex) and hemispheric (cerebral subcortex plus cortex) localization in the brain of rats at 4.5 and 24 h after tMCAO is shown in Fig. 1. Among the 29 animals that were subjected to tMCAO and decapitated at 24 h (IR24 group), 25 and 4 rats exhibited a subcortical and hemispheric localization of the focus, respectively, at 3 h after operation. However, 24 h after occlusion, the number of animals with a subcortical localization decreased to 18 and the number of animals with a hemispheric localization increased to 11. Among the 10 animals that were subjected to tMCAO and decapitated at 4.5 h (IR4.5 group), 8 and 2 rats exhibited a subcortical and hemispheric localization of the focus, respectively. The statistical indicators (the median (Me) and the lower (LQ) and upper (UQ) quartiles for the 25 and 75 percentile interval) of animals from IR24 and IR4.5 groups are shown in Table 1 and Additional file 1, respectively.

**Table 1:** Characterization of ischemia using magnetic resonance imaging

Time after operation (h)	Type of ischemic focus localization	Number of observations	Volume (mm^3^) Me [LQ;UQ]	
			DWI	T2 WI
3	subcortical	25	41.86 [37.97; 53.66]	19.5 [10.93; 28.7]
	hemispheric	4	173.72 [130.31; 208.9]	93.18 [84.37; 95.73]
24	subcortical	18	46.1 [41.6; 56.92]	40.96 [36.06; 51.11]
	hemispheric	11	178.2 [119.01; 201.7]	142.48 [91.72; 173.09]

Statistical indicators (the median (Me) and the lower (LQ) and upper (UQ) quartiles for the 25 and 75 percentile interval) for 29 animals at 3 and 24 h after tMCAO are shown

### Morphology of brain tissues in the experimental groups

**Fig. 2:**
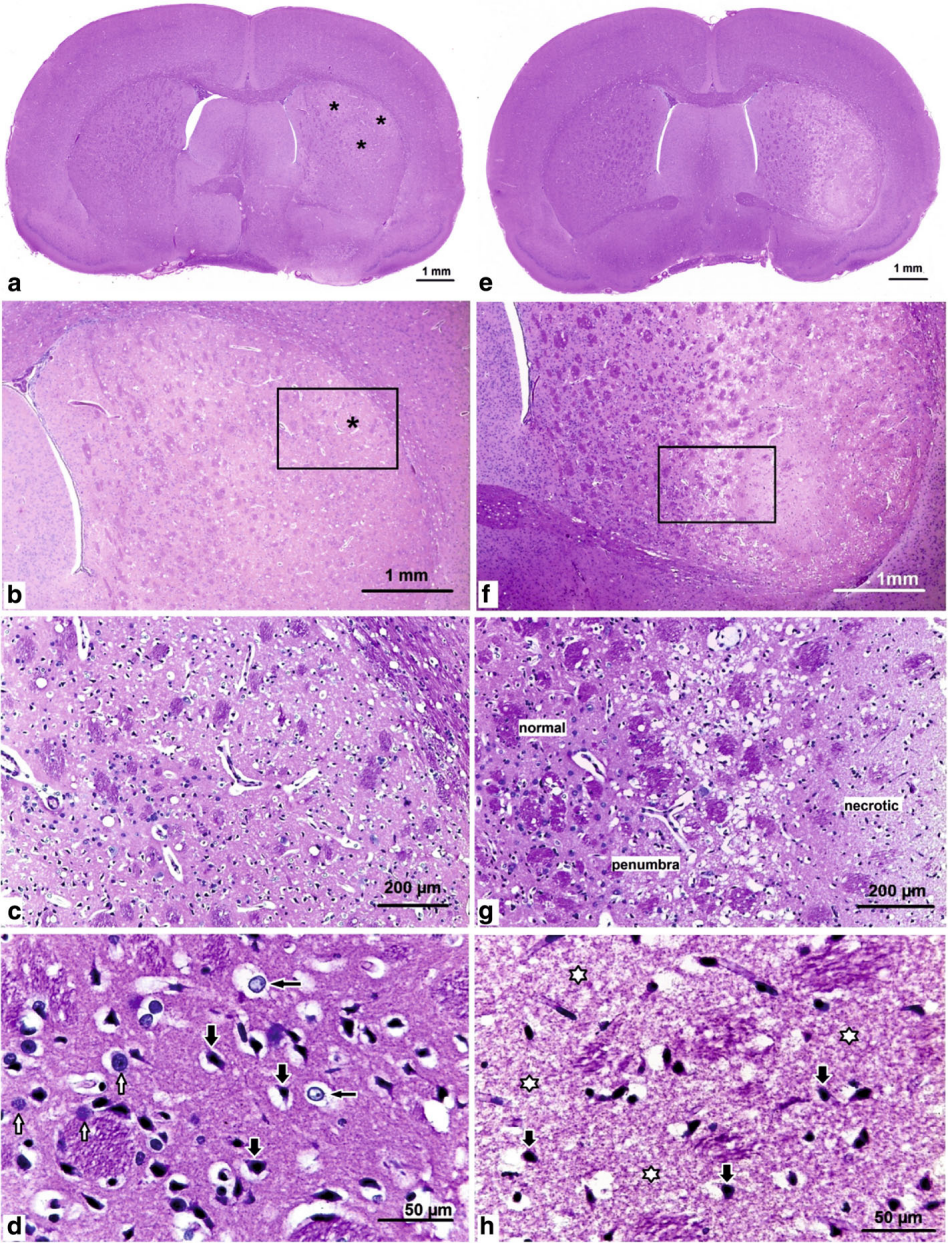
Photomicrographs of haematoxylin and eosin-stained sections of the rat brain in tMCAO model conditions. **a**–**d** 4.5 h after tMCAO. **e**–**h** 24 h after tMCAO. **a**, **e** Coronal rat brain sections at the level of − 0.3 mm from the bregma. Asterisks indicate the damaged area involving the caudoputamen nucleus of the right hemisphere. **b**–**d** High-magnification images of the zone of ischemic injury in the dorsolateral region of the caudoputamen. **c** Area of panel (**b**) marked with a rectangle. **d** Hypoxic damage to neurons, with pyknotic nuclei and peri-cellular oedema indicated in the ischemic zone (thick black arrows); decrease of nuclear basophilia in the neurons (thin black arrows); single intact neurons (white arrows). **f**–**h** Infarction zone within the caudoputamen at higher magnification. **g** Area of panel (**f**) marked with a rectangle. The change zone: from normal through to ischemic-injured (penumbra) to necrotic tissue. **h** Ischemic necrosis of the brain tissue in the central core of an infarct destruction of the neuropil (white asterisks); dead “pyknotic” neurons (thick black arrows)

A typical image of the formation of ischemic injury areas with a subcortical localization in the brain of rats at 4.5 and 24 h after tMCAO is shown in Fig. 2. Focal ischemic lesions of the brain tissue in the form of oedema and foci of “enlightenment” were found in the dorsolateral regions of the subcortical region of the right hemisphere at 4.5 h after tMCAO. The most pronounced changes were observed at − 0.3 mm from the bregma (Fig. 2a, b). Concomitantly, the area of the developing ischemic damage covered a significant area, from the caudoputamen to the corpus callosum and outer capsule. Microscopic examination revealed a drop in the lumen of the capillaries and their emptying, pronounced perivascular oedema, sparsity and bleaching of the neuropil caused by oedema and vacuolization and the appearance of numerous hyperchromic neurons with peri-cellular oedema (Fig. 2c). According to pathomorphological criteria, a significant fraction of neurocytes was in a state of hypoxic damage (reduction of basophilia of chromatin in the nuclei, oedema and homogenization of the cytoplasm) and death (pyknosis and destruction of nuclei and cytoplasmic lysis) (Fig. 2d). The pronounced dystrophic changes observed in the pericarion of most neurons were not detected in the medial region of the caudoputamen outside the zones of the visualized focal ischemia.

The developmental regions of the subcortical infarction zone were observed in the lateral caudoputamen region, from + 1.7 to − 3.3 mm from the bregma, at 24 h after tMCAO. In the range from − 0.3 to − 2.0 mm from the bregma, the infarction zone had an elongated shape in the dorsoventral direction and captured most of the caudoputamen lateral region (Fig. 2e), with distinct visualization of the penumbra and necrosis zones (Fig. 2f, g). The core of the infarction was represented by completely necrotized neural tissue with destruction of all elements of the neuropil and dead “pyknotic” neurons (Fig. 2h). The neurons, both with attributes of ischemic damage and single neurocytes without pronounced pathological changes, were located in the penumbra, the size of which exceeded that of the necrotic zone. The pathological changes were not detected in the medial region of the caudoputamen. In sham-operated animals (SH), the histological pattern of the capillary network and the morphology of neurons in the brain cortex, subcortical centres and the intermediate brain corresponded to the norm.

### RNA-Seq analysis of gene expression profiles in tMCAO model conditions

Using RNA-Seq, the expression level of 17,352 genes was determined in rat brain sub-cortex under tMCAO conditions. Sham-operated animals were used as a control to analyse the mRNA levels of DEGs in the rat sub-cortex after tMCAO at 4.5 h (IR4.5 vs. SH4.5**)** and 24 h after operation (IR24 vs. SH24**)**, respectively. In addition, there were DEGs under sham-operation conditions at 24 vs. 4.5 h after surgical procedure (SH24 vs. SH4.5) and under ischemia–reperfusion conditions at 24 vs. 4.5 h after tMCAO (IR24 vs. IR4.5).

**Fig. 3:**
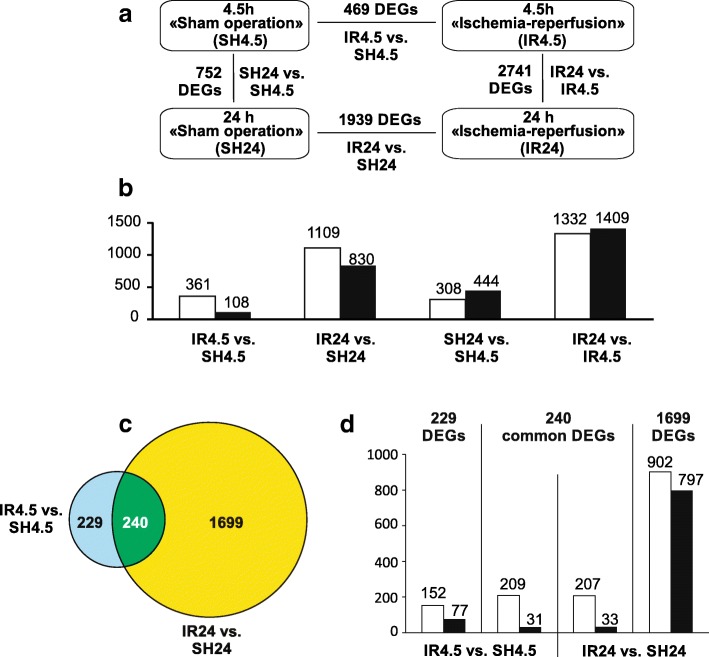
RNA-Seq analysis of differentially expressed genes (DEGs) in tMCAO model conditions. There are RNA-Seq results in IR4.5 vs. SH4.5, IR24 vs. SH24, IR24 vs. IR4.5, SH24 vs. SH4.5. **a** Schematic comparison of the results obtained for the groups. **b** The numbers of up- and down-regulated DEGs are indicated by white and black bars, respectively. The numbers placed above the bars indicate the number of DEGs. **c** The numbers of DEGs overlapped in two pairwise comparisons: IR4.5 vs. SH4.5 and IR24 vs. SH24. **d** Diagrams showing DEGs that lie within the section of sets on the Venn diagrams (**c**). The numbers of up- and down-regulated DEGs are indicated by white and black bars, respectively. The numbers placed above the bars indicate the number of DEGs. Genes with a change in expression > 1.5-fold compared with the baseline value and with a *Padj* < 0.05 were selected for analysis

The number of DEGs in the rat brain sub-cortex in the animal groups studied is shown in the Fig. 3a. There were 469 and 1939 DEGs in IR4.5 vs. SH4.5 and IR24 vs. SH24, respectively. Concomitantly, the number of up-regulated DEGs was greater than the number of down-regulated DEGs at both time points (Fig. 3b). There were 2741 DEGs in IR24 vs. IR4.5. This was the maximum number of DEGs observed between the animal groups studied (Fig. 3b). In particular, we detected an approximately equal number of up-regulated (1332) and down-regulated (1409) DEGs. A similar analysis found 752 DEGs in response to sham operation in SH24 vs. SH4.5, 308 DEGs were up-regulated and 444 down-regulated in this group.

There were 229 genes (152 up- and 77 down-regulated) that changed their expression in IR4.5 vs. SH4.5, but didn’t change it in IR24 vs. SH24 (Fig. 3c). Simultaneously, there were 1699 genes (902 up- and 797 down-regulated) that changed their expression in IR24 vs. SH24, but didn’t change it in IR4.5 vs. SH4.5 (Fig. 3c). All genes predominantly have binding and catalytic activity, but the former DEGs additionally have transcription factor (*Npas4, Fosl2, Fosb*), cytokine (*Ccl2, Ccl3, Il1a, Il1b*), hormone (*Oxt, Avp*) and transporter (*Sec4a5, Kcnj13, Abcg2*) activity, whereas the latter predominantly have cytokine (*Ccl6, Cx3cl1, Il16, Nrtn, Tnfsf9*) and growth factor (*S100a4, Tbrg1, Fgf2*) activity.

We detected 240 common DEGs in the rat sub-cortex between IR4.5 vs. SH4.5 and IR24 vs. SH24 (Fig. 3c). The expression of more than 86% of these DEGs was increased (Fig. 3d). It should be noted that IR had the opposite effect on several genes in IR4.5 vs. SH4.5 and IR24 vs. SH24. In particular, we found 17 up-regulated DEGs (*Nr4a1*, *Nr4a2*, *Nr4a3*, *Egr2*, *Egr3*, *Egr4* and others) and 15 down-regulated DEGs (*Bst2*, *Mx2*, *Ifi27*, *Aldh1a2*, *Xdh* and others) in IR4.5 vs. SH4.5, that exhibited a reversion in the direction of expression in IR24 vs. SH24 (see Additional file 2). The former DEGs predominantly have ligand-activated sequence-specific DNA-binding RNA polymerase II transcription factor activity, whereas the latter predominantly have hydrolase and oxidoreductase activity, as well as receptor-binding and hormone activities.

There were 396 common DEGs in rat sub-cortex between IR24 vs. IR4.5 and SH24 vs. SH4.5 (see Additional file 3: Figure S1A). In particular, 132 DEGs were up-regulated and 264 down-regulated under the sham–operation condition, and 143 DEGs were up-regulated and 253 down-regulated after the development of ischemia–reperfusion damage (see Additional file 3: Figure S1B).

**Fig. 4:**
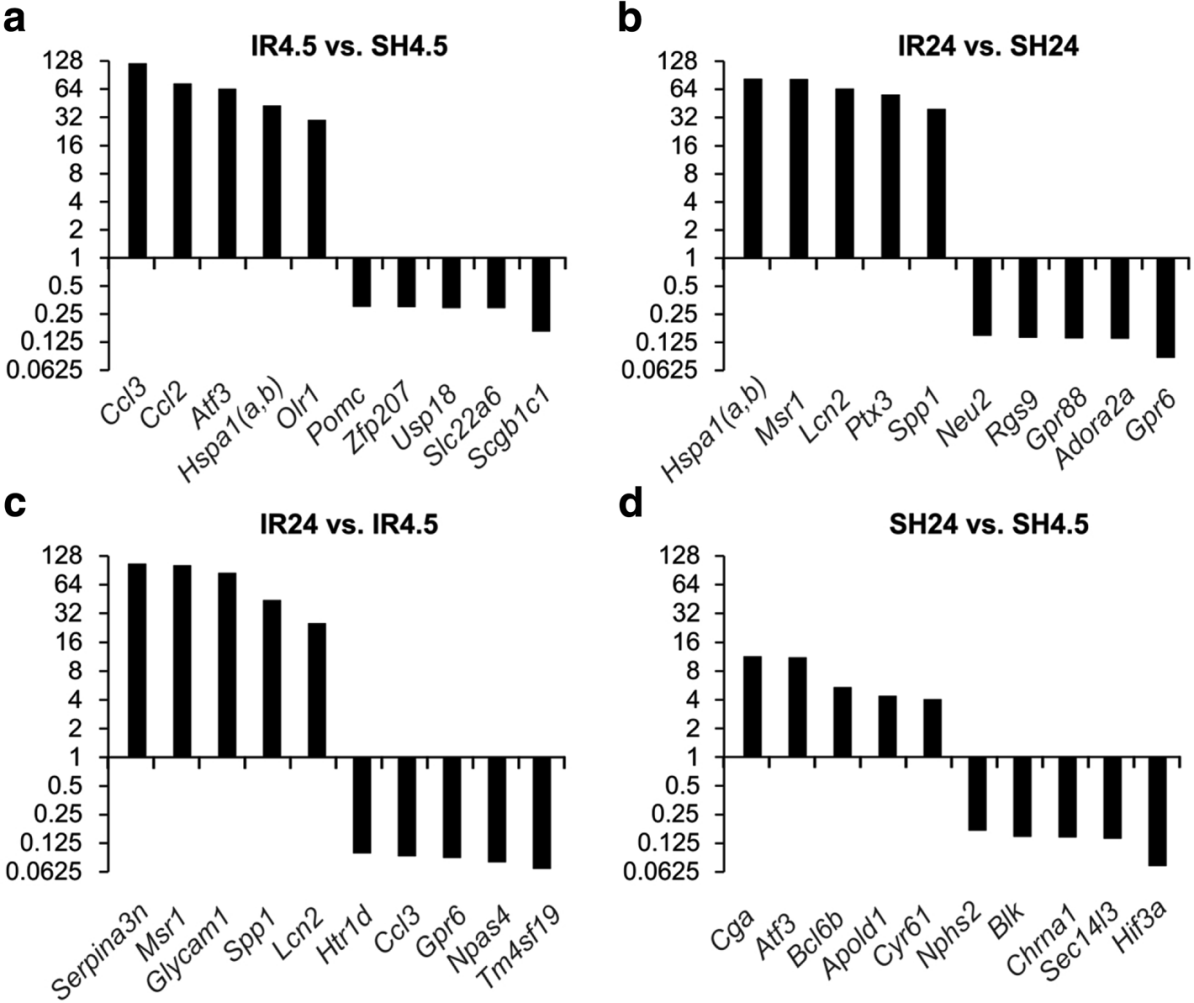
The top five DEGs of each comparison group. There are genes with the greatest fold change in expression in the subcortical structures of the rat brain, as assessed using RNA-Seq. In the comparison of data groups IR4.5 vs. SH4.5 (**a**), IR24 vs. SH24 (**b**), IR24 vs. IR4.5 (**c**) and SH24 vs. SH4.5 (**d**). Differences were considered statistically significant at the probability of *Padj* < 0.05

The top five genes in each of the comparison groups (i.e. those that exhibited the greatest fold change in expression in the subcortical structures of the rat brain) are presented in Fig. 4. The table shows the up-regulation of the mRNA levels of the *Ccl3* and *Ccl2* chemokine genes (> 120-fold and > 70-fold) and those of the transcription factor *Atf3* gene (> 64-fold) at 4.5 h after tMCAO vs. sham operation (Fig. 4a). The mRNA levels of the heat-shock protein *Hspa1(a,b)* gene and *Msr1* gene, which encodes the macrophage scavenger receptor 1, were increased by > 80-fold in IR24 vs. SH24 (Fig. 4b).

The expression of the *Serpina3n* gene, which encodes a serine (or cysteine) peptidase inhibitor, clade A, member 3 N, as well as that of the *Msr1* gene, was increased by > 100-fold in IR24 vs. IR4.5 (Fig. 4c). The fold change of DEGs in SH24 vs. SH4.5 was less than under IR (Fig. 4d).

### Verification of the RNA-Seq results using real-time reverse transcription polymerase chain reaction (RT–PCR)

**Fig. 5:**
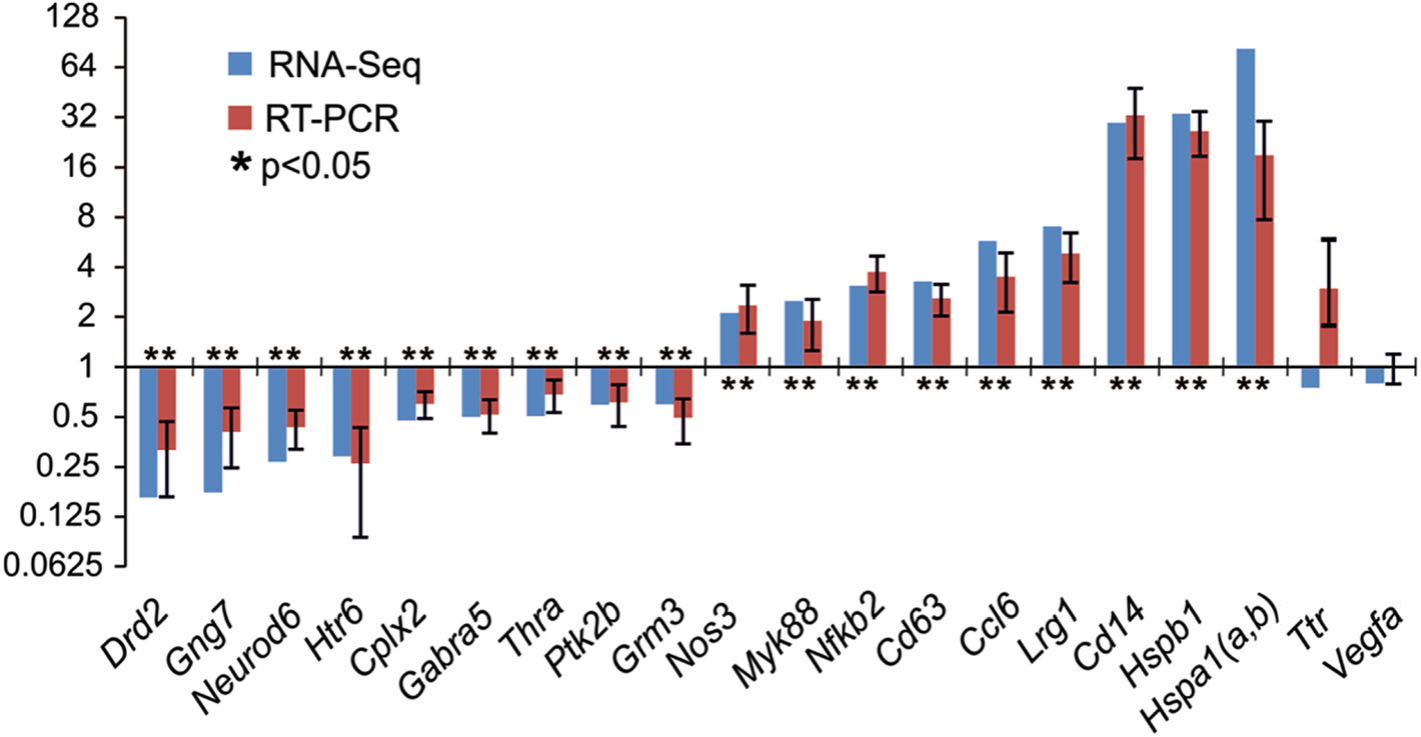
Real-time reverse transcription polymerase chain reaction (RT-PCR) verification of the RNA-Seq results. Data for comparison between the “ischemia–reperfusion” and “sham–operation” groups at 24 h after operation (IR24 vs. SH24) are shown. Two reference genes *Gapdh* and *Rpl3* were used to normalize PCR-results. In each group, there were at least 9 rats. Eighteen genes that change the expression of more than 1.5-fold from the baseline value and whose *P*-value lower 0.05, as well as two other genes were selected for analysis

Real-time reverse transcription polymerase chain reaction (RT–PCR) analysis of the expression of 20 genes was used to verify the RNA-Seq results (Fig. 5). mRNA isolated from the subcortical brain structures of nine rats at 24 h after tMCAO and of nine sham-operated rats was used. Both methods of analysis identified three top genes with a maximum positive expression change (*Hspa1(a,b)*, *Hspb1* and *Cd14*) and three top genes with a maximum negative expression change (*Drd2*, *Gng7* and *Neurod6*). Moreover, both methods identified genes with moderately significant changes in expression levels: up-regulated genes (*Ccl6*, *Cd63*, *Nfkb2*, *Myd88* and *Nos3*) and down-regulated genes (*Grm3*, *Ptk2b*, *Thra*, *Cplx2*, *Gabra5* and *Htr6*). By contrast, significant results regarding the differential expression of the *Ttr* and *Vegfa* genes were not obtained using the two methods (Fig. 5). Thus, the real-time RT–PCR results confirmed the RNA-Seq data sufficiently; the slight variations in gene expression detected by these methods may have been caused by differences in the methodology and statistical processing of data used in each case (Fig. 5).

### Biological processes and functional categories of the proteins encoded by the DEGs identified under the ischemia–reperfusion condition

Analysis of the Gene Ontology database using the PANTHER tool allowed the identification of the biological processes associated with the DEGs identified in the rat brain sub-cortex under the tMCAO model condition. Table 2 shows that the biological processes associated with the DEGs could be divided into 13 functional categories for all comparison groups. The top five categories associated with the largest number of genes included the cellular process, the metabolic process, biological regulation, response to stimuli and localization. The proteins encoded by DEGs were also classified according to their molecular functions. The classification of DEGs according to the molecular functions of their encoded proteins revealed that the largest number of DEGs belonged to the categories of binding, catalytic and transporter activity, as well as signal-transducer and receptor activity (Table 3). The functional categories of the proteins encoded by the DEGs were identified using the David program (see Additional file 4). The damaging effect of IR at 4.5 h after tMCAO affected the expression levels of DEGs associated with 34 functional categories. The total number of functional categories associated with the effects of IR at 24 h after tMCAO exceeded 80 (see Additional file 4), while only six functional categories (phosphoproteins, developmental proteins, biological rhythms, disulphide bonding, glycoproteins and angiogenesis) were associated with SH.

**Table 2:** Analysis of the biological processes associated with the DEGs using the PANTHER tool

Biological Processes	IR4.5 vs. SH4.5	IR24 vs. SH24	IR24 vs. IR4.5	SH24 vs. SH4.5
cellular process (GO:0009987)	189	675	920	245
metabolic process (GO:0008152)	136	450	661	168
biological regulation (GO:0065007)	73	257	326	93
response to stimulus (GO:0050896)	72	226	275	73
localization (GO:0051179)	37	154	192	49
cellular component organization or biogenesis (GO:0071840)	34	151	205	50
developmental process (GO:0032502)	32	139	164	45
multicellular organismal process (GO:0032501)	19	122	167	37
immune system process (GO:0002376)	21	59	51	14
biological adhesion (GO:0022610)	15	44	40	10
locomotion (GO:0040011)	15	37	43	9
reproduction (GO:0000003)	1	10	12	5
rhythmic process (GO:0048511)	2	1	3	3

Associative analysis for all comparison groups was carried out according to the Gene Ontology (GO) database

**Table 3:** Analysis of the molecular functions associated with the DEGs using the PANTHER tool

Molecular Functions	IR4.5 vs. SH4.5	IR24 vs. SH24	IR24 vs. IR4.5	SH24 vs. SH4.5
antioxidant activity (GO:0016209)	0	6	6	2
binding (GO:0005488)	122	409	530	150
catalytic activity (GO:0003824)	99	384	463	124
receptor activity (GO:0004872)	18	77	104	19
signal transducer activity (GO:0004871)	23	71	89	25
structural molecule activity (GO:0005198)	8	50	90	13
transporter activity (GO:0005215)	24	90	140	44
channel regulator activity (GO:0016247)	0	2	2	0
translation regulator activity (GO:0045182)	0	7	10	0

Associative analysis for all comparison groups was carried out according to the Gene Ontology (GO) database

Fifteen functional categories of the proteins encoded by DEGs under IR are presented in Table 4. These were chosen because the expression of the DEGs that encoded these proteins was predominantly changed in each of the categories unidirectionally. Accordingly, the number of up- and down-regulated DEGs is also indicated in Table 4. A detailed analysis of the DEGs detected in each of the comparison groups is presented in Additional files 5, 6, 7, 8, 9, 10, 11, 12, 13, 14, 15, 16, 17, 18, 19. It should be noted that there were up-regulated DEGs associated with the inflammatory, immune and stress responses, as well as with apoptosis and regulation of transcription, in the first hours after the restoration of blood flow in the subcortical structures of the rat brain (see Additional files 5, 6, 10, 11, 16 and 18). Both the number of DEGs associated with the above categories and their level of expression were increased significantly at 24 h after tMCAO compared with the sham-operation conditions. However, a large number of down-regulated DEGs were associated with the transcription process (see Additional file 18). Moreover, dozens of down-regulated DEGs were associated with the functioning of neurotransmitter systems in IR24 vs. SH24 (see Additional files 12 and 17).

**Table 4:** Analysis of the functional categories of the proteins encoded by DEGs

Functional Categories	IR4.5 vs. SH4.5			IR24 vs. SH24			IR24 vs. IR4.5		
	Up	Down	*Padj*	Up	Down	*Padj*	Up	Down	*Padj*
Innate immunity	10	3	5.04E-04	35	3	2.17E-09	30	10	9.38E-07
Inflammatory response	12	0	5.89E-05	16	2	6.39E-03	10	10	3.73E-02
Stress response	7	0	2.80E-03	12	0	1.70E-02			
Differentiation	14	3	3.38E-02	29	24	1.62E-03	31	42	1.58E-04
Cell adhesion	12	4	2.20E-02	29	23	7.52E-05	28	30	6.19E-03
Transcription regulation	35	5	3.54E-04	67	48	1.77E-04	51	108	9.26E-06
DNA replication				14	0	4.54E-03	16	4	1.31E-04
Adaptive immunity				13	4	5.51E-05	11	6	2.20E-03
Apoptosis				38	10	3.38E-04	41	14	9.35E-03
Synapse				2	58	4.10E-07	3	84	1.19E-11
Ion channel				10	43	8.05E-05	17	71	5.76E-12
Proteasome							21	1	1.30E-06
Ribosomal protein							73	4	8.77E-11
Translation regulation							10	3	4.80E-02
Neurogenesis				9	23	1.30E-04	7	29	2.17E-03

Associative analysis of DEGs for comparison groups under ischemia–reperfusion conditions was carried out according to the DAVID database. The number of up-regulated (Up) and down-regulated (Down) DEGs in the condition of the tMCAO model, as well as the *P*-values adjusted using the Benjamini–Hochberg procedure (*Padj*), are shown

The number of DEGs identified in IR24 vs. IR4.5 exceeded that detected in IR24 vs. SH24. In particular, we detected the up-regulation of DEGs that encode proteins associated with the functional categories of DNA replication, proteasome, translation regulation and ribosomal function in IR24 vs. IR4.5 (see Additional files 9, 14, 15 and 19). It should be noted that we detected dozens of down-regulated DEGs that encoded proteins involved in the functioning of neurotransmitter systems and in the regulation of transcription, cell adhesion, differentiation and neurogenesis (see Additional files 7, 8, 12, 13 and 18). Moreover, we identified single DEGs that were associated with the stress response, neurogenesis, transmission of nerve impulses and other processes in the subcortical structures of the rat brain in SH24 vs. SH4.5 (see Additional files 12, 13, 16 and 18).

### Signaling pathways associated with DEGs identified under the ischemia–reperfusion conditions

**Fig. 6:**
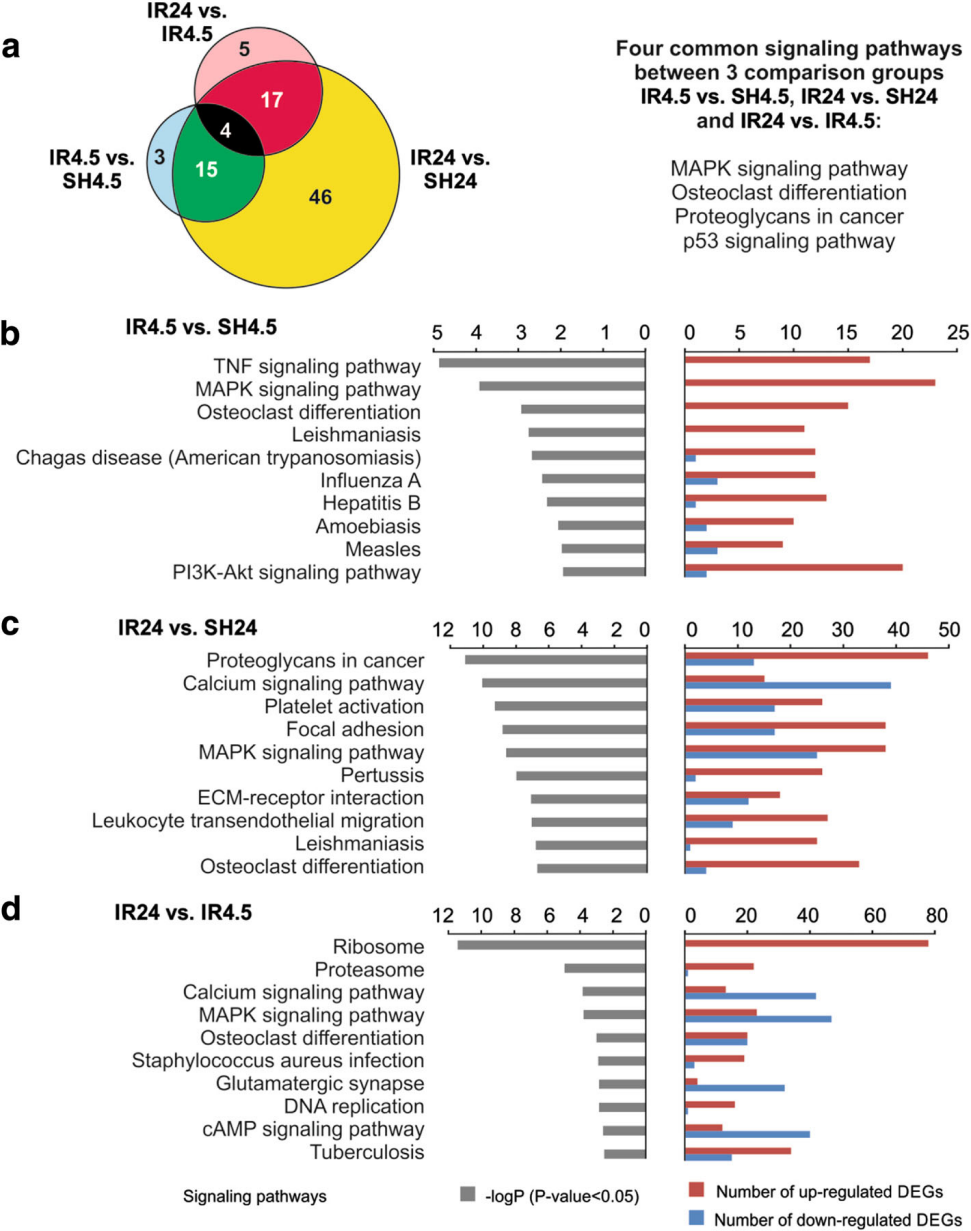
KEGG database analyses of the signaling pathway annotations for differentially expressed genes (DEGs). **a** Venn diagrams showing common and unique signaling pathways associated with DEGs in IR4.5 vs. SH4.5, IR24 vs. SH24, IR24 vs. IR4.5, SH24 vs. SH4.5. The numbers in the sectors indicate the number of signaling pathway annotations for DEGs. **b**, **c**, **d** The most significant signaling pathways and corresponding *P*-values adjusted using the Benjamini–Hochberg procedure, as well as the number of up- and down-regulated DEGs in IR4.5 vs. SH4.5 (**b**), IR24 vs. SH24 (**c**), IR24 vs. IR4.5 (**d**). Genes with a change in expression > 1.5-fold compared with the baseline value and with a *P*-value < 0.05 were selected for analysis

Using DAVID v6.8, a Kyoto Encyclopedia of Genes and Genomes (KEGG) pathway enrichment analysis was used to annotate the functions of the DEGs identified in the rat brain sub-cortex under the IR (see Additional file 20). We found 22 and 82 signaling pathways associated with DEGs in IR4.5 vs. SH4.5 and IR24 vs. SH24, respectively. The former included predominantly signaling pathways involved in the inflammatory response, while the latter included signaling pathways involved in inflammation and neurotransmission. It should be noted that we identified 26 signaling pathways (involved in inflammation, neurosignaling, the formation and functioning of ribosomes, the functioning of proteases, DNA replication and other processes) that were associated with DEGs in the subcortical structure of the rat brain in IR24 vs. IR4.5 (see Additional file 20). A comparative analysis of the signaling pathways identified showed that four signaling pathways that participate in the inflammatory response (MAPK, proteoglycans in cancer, osteoclast differentiation and the p53 signaling pathway) were common to all groups and reflected the effects of IR (Fig. 6a, see Additional file 20). In addition, 15 other common signaling pathways were identified between IR4.5 vs. SH4.5 and IR24 vs. SH24, and 17 common signaling pathways were identified between IR24 vs. IR4.5 and IR24 vs. SH24 groups (Fig. 6a, see Additional file 20). The most significant signaling pathways associated with DEGs in the groups studied are shown in Fig. 6b, c and d. Several signaling pathways involved in the inflammatory response (PI3K-Akt, TNF and others) were associated with DEGs in the rat brain sub-cortex at 4.5 h after tMCAO (Fig. 6b, see Additional file 20). The activation of DEGs associated with focal adhesion, leukocyte trans-endothelial migration, proteoglycan in cancer and other signaling pathways was observed at 24 h after tMCAO (Fig. 6c, see Additional file 20). A comparative analysis of the DEGs detected under the two conditions (IR24 vs. IR4.5 and IR24 vs. SH24) revealed signaling pathways involved in the inflammatory response. However, calcium signaling, glutamatergic synapse, cAMP signaling and other neurosignaling pathways were predominantly associated with DEGs that were down-regulated (Fig. 6d, see Additional file 20). It should be noted that signaling pathways of the ribosome, the proteasome, DNA replication and purine metabolism were predominantly associated with DEGs that were up-regulated in the rat brain sub-cortex in IR24 vs. IR4.5 (see Additional file 20). It should be noted that no reliable association between the DEGs and any signaling pathway was identified in SH24 vs. SH4.5.

## Discussion

Experimental animal models are of great importance for understanding the mechanisms of damage and survival of neurons after ischemic brain injury, as well as for the development of new strategies of neuroprotection. We adapted the tMCAO model, which is used in the development of neuroprotective approaches, for the genome-wide transcriptome analysis of gene expression profiles in the rat brain sub-cortex under conditions of cerebral IR. The main objective of the study was the identification of DEGs, biological processes and signaling pathways associated with the response of brain cells to IR.

MRI and pathomorphological results showed that the specific tMCAO model used in the present study (mode and time of occlusion and reperfusion) allowed the induction of a focal ischemic injury with a predominant subcortical localization. Unlike postmortem triphenyl tetrazolium chloride (TTC) staining, which is used often to localize the area of ischemia [20, 29–31], MRI allowed us to localize the emerging ischemic damage in each animal *in vivo* and to observe the dynamics of its development (Fig. 1). Animals with acute transient impairment of the cerebral circulation manifested their primary lesions as ischemia of the nervous tissues against the background of vascular disturbances. The standard haematoxylin and eosin (HE) staining allowed the verification of changes in the infarct area at the cellular level (Fig. 2).The microscopic changes in the blood supply zone of the right MCA were characterized by the formation of loci of hypoxic damage of neurons with varying severity at 4.5 h after occlusion. Classical zones of formation of an ischemic infarct with deep destructive changes and death of nerve cells in necrotic zones and the large penumbra region were observed at 24 h after occlusion. Thus, MRI and histological examination indicated that the tMCAO model with the characteristics used in the present study led to ischemic injuries with a reproducible localization and volume of ischemic injury, including both the necrosis zone and penumbra. Consequently, the tMCAO model implemented here can be used both for studying the mechanisms of ischemic injury and for testing neuroprotective drugs.

It is well known that cerebral ischemia causes a cascade of biochemical and transcriptome changes in brain tissues [20, 32]. Numerous studies have indicated that early-response genes (*c-fos* and *c-jun*) [33] and zinc finger genes trigger cell proliferation and differentiation [34, 35], while genes that encode heat-shock proteins, are involved in the inflammatory response and cytoskeleton organization [36] and others are predominantly activated after the onset of ischemia. It has been shown that reperfusion after ischemia causes additional damage in brain cells, including destruction of the endothelial cells of the microvascular brain, disturbance of the blood–brain barrier, accumulation of excess oxygen radicals and activation of apoptosis [10, 18, 37–39]. Cerebral ischemic injury caused by artery occlusion in combination with reperfusion leads to numerous disorders of the regulation of mRNAs involved in the cerebral ischemic response [19–21].

We performed a genome-wide analysis of the transcriptome via RNA-Seq in the subcortical structures of the rat brain that contained the ischemic focus and the penumbra region under the conditions of the tMCAO model. Dozens of DEGs were identified using high-throughput RNA-Seq. An analysis of the expression of 20 genes by real-time RT–PCR confirmed the RNA-Seq results. The use of two time points (4.5 and 24 h) for the tMCAO model significantly increased the number of previously known genes involved in the early and late response. We found that 469 and 1939 genes exhibited changes in expression > 1.5-fold at 4.5 and 24 h after tMCAO, respectively. It should be noted that these genes were up-regulated predominantly under the tMCAO model conditions. Among these, we found genes involved in the inflammatory, immune and stress response, as well as in apoptosis and the regulation of transcription (see Additional files 6, 11, 12, 17 and 18). Under IR, the mRNA level of genes encoding chemokines (*Ccl2*, *Ccl3*), heat-shock proteins (*Hspa1(a,b)*), the macrophage scavenger receptor 1 (*Msr1*), the secreted phosphoprotein 1 (*Spp1*) and the suppressor of cytokine signaling 3 (*Socs3*), as well as other genes encoding proteins involved in inflammatory and immune responses, was increased by > 10-fold (Fig. 4). The significant activation of gene expression observed here may indicate the response of living cells in the penumbra to the effects of damage.

Studies with several MRI experiments in rat models of tMCAO demonstrated that stroke-induced infarct evolved into an area of injury, maximal at 24 h [40, 41]. In numerous studies, the molecular mechanisms of IR damage and the effects of neuroprotective agents on the infarct area were studied at 24 h, which is considered the most appropriate time point [19, 20, 27, 42–44]. Wang et al. studied the molecular mechanism of IR pathogenesis using RNA-Seq at 24 h after tMCAO in the hippocampus of rats. Those authors detected 182 DEGs, most of which were up-regulated [20]. A GO analysis showed that these DEGs were mainly associated with inflammation, stress, immune response, glucose metabolism, apoptosis [20]. Our analysis of gene expression under tMCAO conditions using RNA-Seq confirmed these results. However, we found that a > 10-fold greater number of DEGs were associated with a larger number of functional categories and signaling pathways at 24 h after tMCAO. We studied a region of the rat brain that was much larger and included not only the hippocampus but also other subcortical structures that were characterized apparently by a large variety of surviving cells in the peri-infarct region.

We found that 82 signaling pathways were associated with DEGs in IR24 vs. SH24 (see Additional file 20). Among them there were NF-kappa B, TNF, Pathways in cancer, Phagosome, HIF-1, VEGF, Chemokine and other signaling pathways. The significance of the inflammatory signaling pathways (Platelet activation, Focal adhesion, MAPK and others) was increased at 24 h compared with 4.5 h after tMCAO (Fig. 6b, c). In addition, the number of both up-regulated and down-regulated DEGs was increased. It should be noted that among the genes that were down-regulated at 24 h after tMCAO, we found genes involved in the regulation of transcription and the functioning of the neurosignaling pathway (glutamatergic synapse, cholinergic synapse, dopaminergic synapse, calcium signaling pathway and others) (see Additional files 12 and 17).

The analysis of differential gene expression in the rat brain sub-cortex in IR24 vs. IR4.5 and SH24 vs. SH4.5 led to the elucidation of the response of the transcriptome to the development of ischemic injury and to sham operation. It should be noted that the largest number of DEGs (2741) was associated with 26 signaling pathways in IR24 vs. IR4.5. In IR24 vs. IR4.5 we found a significant activation of the expression of genes involved in biosynthetic cell systems (ribosome, proteasome, DNA replication and purine metabolism functional categories), which was not detected in IR4.5 vs. SH4.5 and IR24 vs. SH24 (Fig. 6d, see Additional file 20). The effect obtained indicated a large-scale reorganization of nucleic acid and protein biosynthesis that is apparently related to the adaptive response of brain cells to the damage caused by IR. Concomitantly, between the time points of 4.5 to 24 h after tMCAO, an overwhelming effect of IR on the expression of hundreds of genes involved in the regulation of transcription, cell adhesion, differentiation, neurogenesis and the neurosignaling system was observed (see Additional files 7, 8, 12, 13, 17 and 18). Our results clearly showed that the down-regulation of neurosignaling activity was the consequence of the development of ischemia–reperfusion damage in the tMCAO model. This massive down-regulation of DEGs may be related to the death of cells in the necrotic region or reflect the response of cells of the penumbra to the IR.

The effect of sham operation on gene expression profile in SH24 vs. SH4.5 was highlighted by the differential expression of 752 genes, which included single genes involved in the stress response (*Hspe1*), neurogenesis and differentiation (*Neurod6*), in the response to inflammation (*Cd14*, *Tlr7*) and in several other processes (see Additional files 8, 10, 13 and 16). It should be noted that in SH24 vs. SH4.5 no reliable association was identified between the DEGs and any signaling pathway. However, about 400 common DEGs were found between the SH24 vs. SH4.5 and IR24 vs. IR4.5, at that the direction of their expression change coincided in both cases predominantly. Therefore, it is necessary to consider the contribution of the SH effect when analysing the pathological effect of IR on gene expression in the tMCAO model.

## Conclusions

Thus, the study of the transcriptome profile of cells in the subcortical structures of the brain under tMCAO conditions led to the identification of DEGs that encode proteins that participate in various functional categories, biological processes and signaling pathways, via which brain cells respond to IR. Our results revealed the activation of a large number of genes involved in inflammation, the immune response, apoptosis and the stress response. Simultaneously, under IR, a massive down-regulation of genes that ensure the functioning of neurotransmitter systems was observed. The response of the transcriptome to SH in the condition of the tMCAO model detected here should be considered in studies of the genetic mechanisms underlying the regulation of the response of brain cells to the damaging effects of IR. Therefore, analysis of the transcriptome is one of the most important approaches in a comprehensive study of the effects of the development of ischemic damage. Undoubtedly, this will form the scientific basis of new medical technologies and the development of highly effective drugs to treat this condition.

## Methods

### Animals

White rats of the Wistar line (weight, 200–250 g) were obtained from the Experimental Radiology sector in A. Tsyb Medical Radiological Research Center, Obninsk, Russian Federation. The animals were maintained on a 12 h light/dark cycle at a temperature of 22–24 °C, with free access to food and water. The animals were divided into the “sham operation” (SH) and “ischemia–reperfusion” (IR) groups.

### Transient cerebral ischemia rat model

The transient cerebral ischemia rat model was induced by endovascular occlusion of the right middle cerebral artery using a monofilament (Doccol Corporation, USA) for 90 min, and subsequent reperfusion for 3 or 22.5 h using the method of Koizumi et al., [45] with modifications. The rats were decapitated at 4.5 or 24 h after tMCAO (group “IR4.5” or “IR24”, respectively). Prior to the surgical procedure, rats were anesthetized using 3% isoflurane; the anaesthesia was maintained using 1.5–2% isoflurane and the EZ–7000 small animal anaesthesia system (E-Z Anesthesia, USA). The sham-operated rats (groups “SH4.5” and “SH24”, respectively) were subjected to a similar surgical procedure under anaesthesia (neck incision and separation of the bifurcation), but without tMCAO. Each experimental group consisted of at least fifteen animals. The subcortical structures were separated from the rat brain. Tissues were placed in RNAlater solution for 24 h at 0 °C and then stored at − 70 °C.

### MRI

The MRI study of the characteristics and size of the ischemic injury of rat brains was carried out using small animal 7 T ClinScan tomograph (Bruker BioSpin, USA). The standard protocol included the following modes: DWI with mapping of the apparent diffusion coefficient for assessing acute ischemic damage, and T2 WI in the transversal plane. Magnetic resonance angiography (3D-TOF MRA) was used for visualization of the main arteries and control of the recanalization. Quantitative assessment of the volume of the infarction focus was performed using the ImageJ software package (Wayne Rasband, National Institute of Mental Health, Bethesda, MD, USA). MRI was performed immediately before decapitation in rats from the IR4.5 group. In rats from the IR24 group, MRI was performed twice: at 3 h after tMCAO and immediately before decapitation.

### Histological examination of rat brains

Tissue samples of rat brains from each group (IR4.5, IR24, SH4.5 and SH24; *n* = 3–4) were immersed in Buin’s fluid for 24 h and washed with 70% ethanol. Tissue samples were dehydrated and embedded in Histomix® (BioVitrum, Russia). Tissue sectioning was performed with the orientation of two tissue blocks for subsequent excision into coronary sections at the level from − 4.0 to − 0.5 and from − 0.5 to + 5 mm from the bregma. Sections with a thickness of 5–6 μm obtained through 0.5–1 mm on a microtome (Leica RM2235, Germany) were stained with haematoxylin and eosin (BioVitrum) after dewaxing. Histological specimens were examined under a microscope (Leica DM 1000) with a micrograph to digital camera (Leica ICC50 HD). Morphological analysis was performed with allowance for normal and pathological central nervous system variants [46–48]. Stereotactic mapping of the damaged zones and accurate determination of the level of sections were performed according to an atlas of the rat brain.

### RNA isolation

Total RNA from the subcortex, was isolated using TRIzol reagent (Invitrogen, Thermo Fisher Scientific) and acid guanidinium thiocyanate–phenol–chloroform extraction [49]. The isolated RNA was treated with deoxyribonuclease I (DNase I) (Thermo Fisher Scientific) in the presence of RiboLock ribonuclease (RNase) inhibitor (Thermo Fisher Scientific), according to the manufacturer’s recommended protocol. Deproteinization was performed using a 1:1 phenol:chloroform mixture. The isolated RNA was precipitated with sodium acetate (3.0 M, pH 5.2) and ethanol. RNA integrity was checked using capillary electrophoresis (Experion, BioRad, USA).

### RNA-Seq

Total RNA isolated from the subcortical structures of the brain, including the lesion focus, was used in this experiment. The RNA-Seq experiment was conducted with the participation of ZAO Genoanalytika, Russia. For RNA-Seq, the polyA fraction of the total RNA was obtained using the oligoT magnetic beads of the Dynabeads® mRNA Purification Kit (Ambion, USA). cDNA (DNA complementary to RNA) libraries were prepared using the NEBNext® mRNA Library Prep Reagent Set (NEB, USA). The concentration of cDNA libraries was measured using Qbit 2.0 and the Qubit dsDNA HS Assay Kit (Thermo Fisher Scientific, USA). The length distribution of library fragments was determined using the Agilent High Sensitivity DNA Kit (Agilent, USA). Sequencing was carried out using an Illumina HiSeq 1500 instrument. At least 10 million reads (1/50 nt) were generated.

### RNA-Seq data analysis

Four variants of the comparisons of RNA-Seq results were used to identify DEGs under tMCAO model conditions. Two variants showed DEGs under ischemia–reperfusion after tMCAO at 4.5 h vs. sham operation at 4.5 h after surgical procedure (IR4.5 vs. SH4.5) and under ischemia–reperfusion after tMCAO at 24 h vs. sham operation at 24 h after surgical procedure (IR24 vs. SH24). In addition, there were DEGs. Two other variants identified DEGs under sham-operation conditions at 24 vs. 4.5 h after surgical procedure (SH24 vs. SH4.5) and under ischemia–reperfusion conditions at 24 vs. 4.5 h after tMCAO (IR24 vs. IR4.5). Each of the comparison groups (IR4.5, IR24, SH4.5 and SH24) included three animals. To obtain the most representative results, the RNA-Seq analysis was performed using brain samples of rats with a subcortical localization of the ischemic focus exclusively. All genes were annotated on the NCBI Reference Sequence database. The levels of gene expression were measured as fragments per kilobase per million reads using the Cuffdiff program. Only genes that exhibited changes in expression > 1.5-fold and had a *P*-values adjusted using the Benjamini–Hochberg procedure lower 0.05 (*Padj* < 0.05) were considered.

### cDNA synthesis

cDNA synthesis was conducted in 20 μl of reaction mixture containing 2 mg of RNA using the reagents of a RevertAid First Strand cDNA Synthesis Kit (Thermo Fisher Scientific) in accordance with the manufacturer’s instructions. Oligo (dT)_18_ primers were used to analyse mRNA.

### Real-time RT–PCR

The 25 μl PCR mixture contained 2 μl of 0.2× reverse transcriptase reaction sample, forward and reverse primers (5 pmol each), 5 μl of 5× reaction mixture (Evrogen Joint Stock Company) including PCR buffer, Taq DNA polymerase, deoxyribonucleoside triphosphates (dNTP) and the intercalating dye SYBR Green I. Primers specific to the genes studied were selected using OLIGO Primer Analysis Software version 6.31 and were synthesized by the Evrogen Joint Stock Company (see Additional file 21). The amplification of cDNAs was performed using a StepOnePlus Real-Time PCR System (Applied Biosystems, USA) in the following mode: stage 1 (denaturation), 95 °C, 10 min; stage 2 (amplification with fluorescence measured), 95 °C, 1 min; 65 °C, 1 min; 72 °C, 1 min (40 cycles).

### Data analysis of real-time RT–PCR and statistics

Two reference genes *Gapdh* and *Rpl3* were used to normalize the cDNA samples [50]. Calculations were performed using BestKeeper, version 1 [51] and Relative Expression Software Tool (REST) 2005 software [52]. The manual at the site ‘REST.-gene-quantification.info’ (http://www.gene-quantification.de/rest-2009.html) was used to evaluate expression target genes relative to the expression levels of the reference genes. The values were calculated as Ef^Ct(ref)^/Ef^Ct(tar)^, where Ef is the PCR efficiency, Ct(tar) is the average threshold cycle (Ct) of the target gene, Ct(ref) is the average Ct of the reference gene, and Ef^Ct(ref)^ is the geometric average Ef^Ct^ of the reference genes. PCR efficiencies were assessed using the amplification of a series of standard dilutions of cDNAs and computed using REST software [52]. The efficiency values for all PCR reactions were in the range 1.83 to 2.08 (see Additional file 21). At least 9 animals were included in each comparison group. When comparing data groups, statistically significant differences were considered with the probability *p* < 0.05. Additional calculations were performed using Microsoft Excel.

### Functional analysis

Database for Annotation, Visualization and Integrated Discovery (DAVID v6.8, https://david.ncifcrf.gov/tools.jsp) [53] and The PANTHER database (Protein ANalysis THrough Evolutionary Relationships, http://pantherdb.org) [54] were used to annotate the functions of the differentially expressed genes. When comparing data groups, statistically significant differences were considered with the probability *P* < 0.05. To control the false discovery rate we used Benjamini–Hochberg procedure.

## Additional files

Additional file 1: **Table S1.** Characterization of ischemia at 4.5 after tMCAO using DWI mode of MRI. (XLSX 9 kb)

Additional file 2: **Table S2.** DEGs that exhibited a reversed direction of expression in IR4.5 vs. SH4.5 and IR24 vs. SH24. (XLSX 13 kb)

Additional file 3: **Figure S1.** RNA-Seq analysis of differentially expressed genes (DEGs) in tMCAO model conditions. (PPTX 2306 kb)

Additional file 4: **Table S4.** Analysis of the functional categories of the proteins encoded by DEGs. (XLSX 21 kb)

Additional file 5: **Table S5.** The RNA-Seq analysis of DEGs associated with the functional category “Adaptive immunity”. (XLSX 13 kb)

Additional file 6: **Table S6.** The RNA-Seq analysis of DEGs associated with the functional category “Apoptosis”. (XLSX 23 kb)

Additional file 7: **Table S7.** The RNA-Seq analysis of DEGs associated with the functional category “Cell adhesion”. (XLSX 23 kb)

Additional file 8: **Table S8.** The RNA-Seq analysis of DEGs associated with the functional category “Differentiation”. (XLSX 28 kb)

Additional file 9: **Table S9.** The RNA-Seq analysis of DEGs associated with the functional category “DNA replication”. (XLSX 15 kb)

Additional file 10: **Table S10.** The RNA-Seq analysis of DEGs associated with the functional category “Inflammatory response”. (XLSX 14 kb)

Additional file 11: **Table S11.** The RNA-Seq analysis of DEGs associated with the functional category “Innate immunity”. (XLSX 34 kb)

Additional file 12: **Table S12.** The RNA-Seq analysis of DEGs associated with the functional category “Ion channel”. (XLSX 28 kb)

Additional file 13: **Table S13.** The RNA-Seq analysis of DEGs associated with the functional category “Neurogenesis”. (XLSX 20 kb)

Additional file 14: **Table S14.** The RNA-Seq analysis of DEGs associated with the functional category “Proteasome”. (XLSX 13 kb)

Additional file 15: **Table S15.** The RNA-Seq analysis of DEGs associated with the functional category “Ribosomal protein”. (XLSX 23 kb)

Additional file 16: **Table S16.** The RNA-Seq analysis of DEGs associated with the functional category “Stress response”. (XLSX 17 kb)

Additional file 17: **Table S17.** The RNA-Seq analysis of DEGs associated with the functional category “Synapse”. (XLSX 29 kb)

Additional file 18: **Table S18.** The RNA-Seq analysis of DEGs associated with the functional category “Transcription regulation”. (XLSX 49 kb)

Additional file 19: **Table S19.** The RNA-Seq analysis of DEGs associated with the functional category “Translation regulation”. (XLSX 12 kb)

Additional file 20: **Table S20.** Analysis of the signaling pathways associated with DEGs in the condition of the tMCAO model. (XLSX 19 kb)

Additional file 21: **Table S21.** The characterization of the primers for real-time RT-PCR. (XLSX 12 kb)

## Abbreviations

3D-TOF MRA: Magnetic resonance angiography
cDNA: DNA Complementary to RNA
Ct: Threshold cycle
DEGs: Differentially expressed genes
DNase: Deoxyribonuclease
dNTP: Deoxyribonucleoside triphosphate
DWI: Diffusion-weighted imaging
Ef: The PCR efficiency
HE: Haematoxylin and eosin
IR: Ischemia–reperfusion conditions
IR24: Ischemia–reperfusion at 24 h after tMCAO
IR4.5: Ischemia–reperfusion at 4.5 h after tMCAO
KEGG: Kyoto Encyclopedia of Genes and Genomes
LQ: The lower quartiles for the 25 percentile interval
Me: The median
MRI: Magnetic resonance imaging
nt: Nucleotide(s)
*Padj*: *p*-values adjusted using the Benjamini–Hochberg procedure
REST: Relative Expression Software Tool
RNase: Ribonuclease
RNA-Seq: High-throughput RNA sequencing
RT–PCR: Reverse transcription polymerase chain reaction
SH: Sham-operation conditions
SH24: Sham-operation conditions at 24 h after surgical procedure
SH4.5: Sham-operation conditions at 4.5 h after surgical procedure
T2 WI: T2–weighted imaging
tMCAO: Transient middle cerebral artery occlusion
TTC: Triphenyl tetrazolium chloride
UQ: The upper quartiles for the 75 percentile interval
