# Supplementary material for: Genome-wide transcriptome analysis using RNA-Seq reveals a large number of differentially expressed genes in a transient MCAO rat model
Source: BMC Genomics. 2018 Sep 5;19:655. doi: 10.1186/s12864-018-5039-5 (PMC6125876; doi:10.1186/s12864-018-5039-5)
Supplement: Supplementary file 3 — Figure S1. RNA-Seq analysis of differentially expressed genes (DEGs) in tMCAO model conditions. (PPTX 2306 kb) [file 12864_2018_5039_MOESM3_ESM.pptx]

## Slide 1
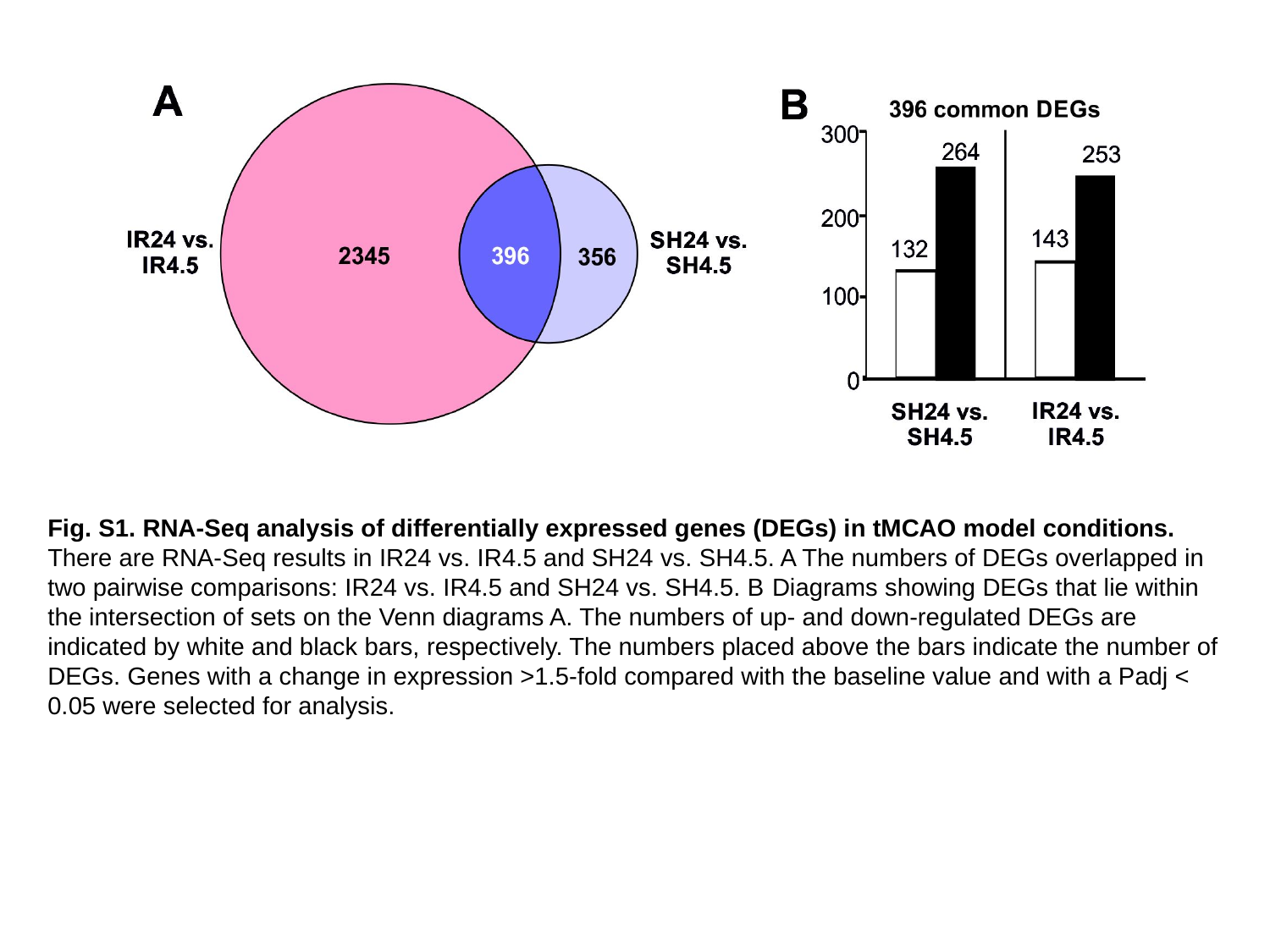

Fig. S1. RNA-Seq analysis of differentially expressed genes (DEGs) in tMCAO model conditions. There are RNA-Seq results in IR24 vs. IR4.5 and SH24 vs. SH4.5. A The numbers of DEGs overlapped in two pairwise comparisons: IR24 vs. IR4.5 and SH24 vs. SH4.5. B Diagrams showing DEGs that lie within the intersection of sets on the Venn diagrams A. The numbers of up- and down-regulated DEGs are indicated by white and black bars, respectively. The numbers placed above the bars indicate the number of DEGs. Genes with a change in expression >1.5-fold compared with the baseline value and with a Padj < 0.05 were selected for analysis.
